# Fine Particulate Matter Exposure and Risk of Major Adverse Cardiac and Cerebrovascular Events (MACCE) in Post-Percutaneous Coronary Intervention (PCI) Patients: A Thai PCI Registry-Based Cohort Study

**DOI:** 10.5334/gh.1539

**Published:** 2026-03-17

**Authors:** Chaiyawat Suppasilp, Teeranan Angkananard, Romen Samuel Rodis Wabina, Worawut Roongsangmanoon, Pawin Numthavaj, Phunchai Charatcharoenwitthaya, Atiporn Ingsathit, Kriengsak Vareesangthip, Suphot Srimahachota, Thosapol Limpijankit, Nakarin Sansanayudh, Ammarin Thakkinstian

**Affiliations:** 1 Department of Clinical Epidemiology and Biostatistics, Faculty of Medicine Ramathibodi Hospital, Mahidol University, Bangkok, Thailand; 2 Division of Cardiovascular Medicine, Department of Medicine, Faculty of Medicine, HRH Princess Maha Chakri Sirindhorn Medical Center, Srinakharinwirot University, Nakhon Nayok, Thailand; 3 Division of Gastroenterology, Department of Medicine, Faculty of Medicine, Siriraj Hospital, Mahidol University, Bangkok, Thailand; 4 Renal Division, Department of Medicine, Faculty of Medicine Siriraj Hospital, Mahidol University, Bangkok, Thailand; 5 Division of cardiovascular Medicine, Department of Medicine, King Chulalongkorn Memorial Hospital, Thailand; 6 Division of Cardiovascular Medicine, Department of Medicine, Faculty of Medicine, Ramathibodi Hospital, Mahidol University, Bangkok, Thailand; 7 Division of Cardiovascular Medicine, Department of Medicine, Phramongkutklao Hospital, Bangkok, Thailand

**Keywords:** fine particulate matter, PM2.5, percutaneous coronary intervention, cardiovascular events, coronary artery disease

## Abstract

**Background:**

Major adverse cardiac and cerebrovascular events (MACCE) are critical clinical outcomes in patients undergoing percutaneous coronary intervention (PCI); however, evidence regarding the impact of fine particulate matter (PM2.5) on these outcomes remains limited.

**Methods:**

This retrospective cohort study included 22,188 Thai adults who underwent PCI to investigate the association between PM2.5 exposure and the incidence of MACCE. Baseline demographic, clinical characteristics, and comorbidities, with angiographic and procedural data, were collected. Cumulative PM2.5 exposure was estimated using satellite-derived data based on patients’ residential locations over a 12-month follow-up period. The primary outcome was a composite MACCE endpoint. A multilevel survival model was employed to assess the association between PM2.5 exposure and MACCE, adjusting for potential confounding variables.

**Results:**

During the median follow-up of 11.97 months (ranging from 0.03 to 12 months), 6,382 patients (28.8%) experienced at least one MACCE. PM2.5 levels in Thailand exhibit a distinct seasonal pattern, peaking around February (Quarter 1; Q1) and reaching their lowest levels in Q3. In the final multivariable model, a 1 µg/m^3^ increase in PM2.5 exposure was associated with MACCE (adjusted hazard ratio (HR) 1.45 (95% CI: 1.37, 1.54)). The adjusted HR for PM2.5 comprising quarterly seasonal variations was as follows: 1.015 (95% CI: 1.005, 1.024) in Q4, 1.222 (95% CI: 1.132, 1.319) in Q1, 1.177 (95% CI: 1.096, 1.265) in Q2, and 1.500 (95% CI: 1.381, 1.629) in Q3.

**Conclusion:**

The study’s findings suggested that higher seasonal PM2.5 exposure is associated with MACCE in patients who underwent PCI. These results underscore the urgent need for public health policies that focus on reducing PM2.5 to improve health outcomes and reduce the burden of the disease.

## Graphical Abstract

**Figure:**
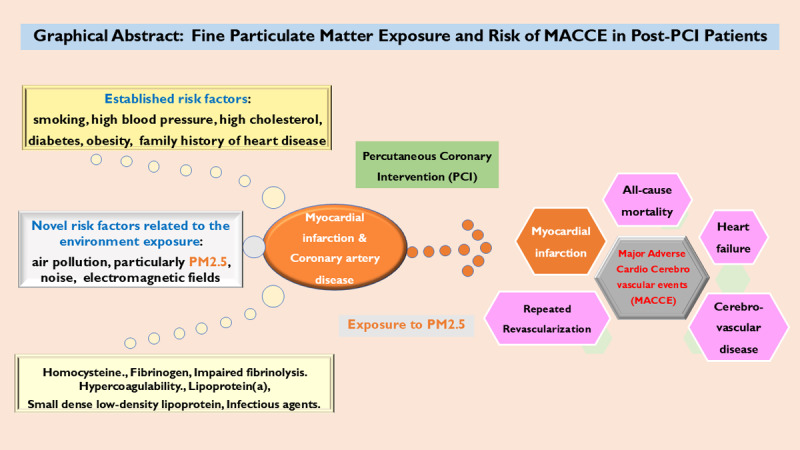


## Introduction

Cardiovascular diseases (CVD) impact a vast majority of people worldwide, with ischemic heart disease being the leading cause of death in the global population, accounting for 19.05 million deaths in 2020, and high economic cost (1). Known contributing factors of CVD include hypertension (HT), diabetes mellitus (DM), dyslipidemia (DLP), and obesity. Apart from clinical risk factors, behavioral factors, including social, cultural, and environmental aspects, also play a role in determining the severity and complications of CVD (2). Due to the significant impact of CVD on healthcare, determining risk factors and how to minimize them could contribute to reducing its burden. Coronary heart disease (CHD) is estimated to affect 20.5 million people in the United States (1). About 1 in 20 adults age 20 and older have coronary artery disease (CAD), in which acute coronary syndrome (ACS) was estimated to be responsible for one-third of total deaths in people older than 35 (3). In Thailand, the Ministry of Public Health has reported an increasing incidence of CHD each year, with mortality rate of 21,874 people (33.54 per 100,000 population) for the year 2021 (4).

Pollution is one of the largest environmental causes of disease and premature deaths worldwide, with an estimated 8.34 million [95% confidence interval (CI) 5.63 to 11.19] global all-cause attributable deaths per year (5) and four to ten million deaths per year from outdoor (ambient) particulate pollution attributed to fine particulate matter (particulate matter with a diameter of less than 2.5 microns; PM2.5) (6). In many parts of the world, ambient air pollution and chemical pollution are rapidly increasing, especially in low- and middle-income countries. The risk of all non-communicable diseases, including CVD, increases as exposure to air pollution increases (7). This includes relationships between risk of disease and exposure to airborne fine particulates in short- and long-term exposure (8). PM2.5 can deposit at the terminal respiratory airways and alveoli (9) and increase the risk of CV mortality (8,10). Short-term elevations of PM2.5 can increase relative risks of acute CV events within one day’s exposure (11,12) and the same effect was shown in patients with longer exposure (13,14). The risk for ST-elevation myocardial infarction (STEMI), but not non-ST elevation myocardial infarction (NSTEMI), may be more closely associated with short-term PM2.5 exposure at different time points before a CV event (11,15).

Robust epidemiological evidence has consistently linked both short-term and long-term PM2.5 exposure to increased hospitalizations for myocardial infarction (16). Furthermore, long-term exposure to PM2.5 is positively associated with increased stroke incidence (17). Mechanistically, PM exposure has been shown to induce cardiac arrhythmias via the impairment of ion channels, a phenomenon corroborated by findings in both clinical and laboratory studies (18). Adverse associations between general air pollution and hospitalizations for heart failure (HF) have also been widely reported, with consistent effects observed across both short-term and long-term exposure durations (19,20).

Cardiometabolic risk factors, such as HT and insulin resistance, were associated with PM2.5 exposure (21). Annual mean PM2.5 concentrations in Thailand reached 24.3, 21.4, 20.2, 18.1, and 23.3 μg/m^3^ from 2019 to 2023 (22), ranking it the 28^th^ most polluted country in 2019. Estimated annual all-cause mortality and cardiovascular deaths attributable to PM2.5 of the Thai population were about 38,410 and 15,361 deaths, respectively (23). Persistently high concentrations of PM2.5 are caused by smoke emissions from forest fires, which predominate in the North and Northeast regions, and the effects of agricultural burning in the Central region of Thailand (24). Adults with preexisting CVD undergoing elective percutaneous coronary intervention (PCI) could be vulnerable to PM2.5-related CVD mortality (25,26,27), yet data on its impact on other CV events in the PCI population are limited. Clarifying this relationship could inform preventive strategies, particularly for short-term exposure, which may be more modifiable. In addition, an interplay between CVD and SARS-CoV-2 infection was noted, which may increase the risk of CVD and modify its risk factors, such as HT or DM, and increas CVD incidence and worsen clinical outcomes in individuals with preexisting CVD (28,27,29). This study has investigated the association between PM2.5 exposure and composite CV events, including major adverse cardiac and cerebrovascular events (MACCE), in Thai adults undergoing PCI.

## Material and Methods

### Subjects and settings

This retrospective cohort study was conducted using data from the Thai PCI Registry, including patients who underwent PCI between May 2018 and July 2019. (30,31). Patients were included with the following criteria: 1) Adults aged 18 years or older, 2) Have undergone PCI at any of 39 hospitals across the country 3) Have been prescribed dual-antiplatelets therapy for at least 3 months after PCI, and 4) could be discharged after the initial PCI procedure. They were excluded if they were lost to follow-up and had received incomplete therapy for 1 year after ACS events, including NSTEMI or unstable angina and STEMI, no residential address that could be mapped with the areas of PM2.5 exposure; and had stent thrombosis, recurrent MI, or death during initial PCI hospitalization.

This study was approved by the Institutional Review Board Committee of Srinakharinwirot University (SWUEC-192/2564E), written informed consent was obtained from each patient included in the study, and the study protocol has conformed with the ethical guidelines of the 1975 Declaration of Helsinki.

### PM2.5 exposure

PM2.5 data was prepared by the Geo-informatics and Space Technology Development Agency of Thailand (GISTDA) using the outputs from the geostationary satellite data, Himawari-8. The Himawari-8-AHI Aerosol Optical Depth (AOD) Level-3 with a temporal resolution of 10 minutes and a spatial resolution of 0.05 × 0.05 degrees was used for separate development of seasonal equations (winter, summer, and rainy). Additionally, the land model accounted for different land types, and the atmospheric model accounted for meteorology factors (e.g., wind speed, humidity, and temperature), which were separately developed to compensate for the incompleteness of PM2.5 estimation in every needed grid. In addition, PM2.5 data measured directly by monitoring stations of the Pollution Control Department of the Ministry of Natural Resources and Environment of Thailand and AERONET of the National Aeronautics and Space Administration (NASA) were used for cross-validation. Finally, continuous grid-based data were aggregated using zonal statistics to obtain hourly values for each sub-district. These 24-hourly values were then averaged to derive the daily mean for each subdistrict.

### Outcome of interest and covariates

The outcome of interest was MACCE, which comprised myocardial infarction (MI), HF, cerebrovascular accident (CVA, including stroke and transient ischemic attack), repeated unplanned revascularization, and all-cause death. In alignment with the registry protocol, clinical outcomes were assessed at predefined 6- and 12-month post-discharge milestones; however, event data were retrospectively recorded using the precise dates of occurrence. The date of each individual outcome was separately recorded with a one-year follow-up period after cohort enrollment, after which it was right-censored.

This study measured possible covariates, which included age, gender, body mass index (BMI), comorbidity of HT, DM, DLP, chronic kidney disease (CKD), previous MI, family history of premature CVD, prior PCI, prior Coronary Artery Bypass Grafting (CABG), and smoking. Moreover, clinical data was obtained, including types of CAD, HF, arrhythmia, and cardiogenic shock at initial presentation. Data from the PCI procedure was also obtained, including the number of lesions, the number of stents used, and left main (LM) vessel involvement.

The COVID-19 pandemic period from January 2020 to December 2021 was modeled as a time-varying dummy covariate in the survival analyses. This period represented the time when hospitals in Thailand faced insufficient healthcare resources and inefficient workforce allocation, focusing more on these issues than on the direct clinical effects of COVID-19 infection itself.

### Data preparation

Individual patient data from the PCI registry were structured on a monthly basis to accommodate time-varying exposure and covariates while preserving exact event timing. Each patient contributed up to 12 observations, unless censored due to death or a loss of follow-up. Each monthly record captured the occurrence of MACCE (yes/no) and its corresponding exact event date, alongside time-varying covariates. If there was no event in a month, the last day of the month was the last follow-up date. Monthly average PM2.5 concentrations were linked to each record based on the patient’s residential sub-district and the dates corresponding to that month. In cases where a MACCE event occurred mid-month, the average PM2.5 exposure was calculated using only the days preceding the event for that month; the remaining days were attributed to the subsequent month’s exposure value. Seasonality was addressed analytically using calendar-quarter indicator variables rather than by aggregating data at the quarterly level, allowing finer temporal alignment between PM2.5 exposure and outcome occurrence while accounting for seasonal variation.

### Statistical Analyses

Categorical variables are shown as frequencies and percentages, and continuous variables are expressed as the mean with standard deviation (SD) or the median with interquartile range (IQR), depending on the data distribution. These corresponding data were compared between groups with and without MACCE, using the chi-square and Student’s t-test or Mann-Whitney U test, where appropriate.

Two-level (district and individual) mixed-effect parametric Weibull survival regression in an accelerated failure time (AFT) framework was employed to assess the association between PM2.5 exposure, as a continuous variable, and MACCE outcome with the following steps: First, a univariable analysis was performed by fitting each of PM2.5 exposure and covariates in the model. Second, variables with a p-value less than 0.20 or a Hazard Ratio (HR) > 1.20 (or not < 0.83) in this step were simultaneously considered in the multivariable model containing PM 2.5. Backward elimination was utilized by applying a likelihood-ratio test to select only significant variables in the final model. The final multivariable model was selected after evaluating potential interaction effects between PM2.5 exposure and the calendar quarter of the year. Although the data were organized on a monthly basis, seasonal variation was addressed by incorporating calendar-quarter dummy variables into the models. An HR along with 95% confidence interval (CI) was then estimated accordingly.

Subgroup analyses were conducted to assess the effect of PM2.5 on the outcome across different types of CAD, including stable CAD, NSTEMI/unstable angina, and STEMI, as well as across regions with differing levels of wildfire activity (high: northern, central, and northeastern regions; low: all remaining regions).

All analyses were conducted using Stata 18 software (StataCorp.2023. Stata Statistical Software: Release 18. College Station, TX: StataCorp LLC). A p-value of less than 0.05 was considered as statistical significance.

## Results

**Figure 1:**
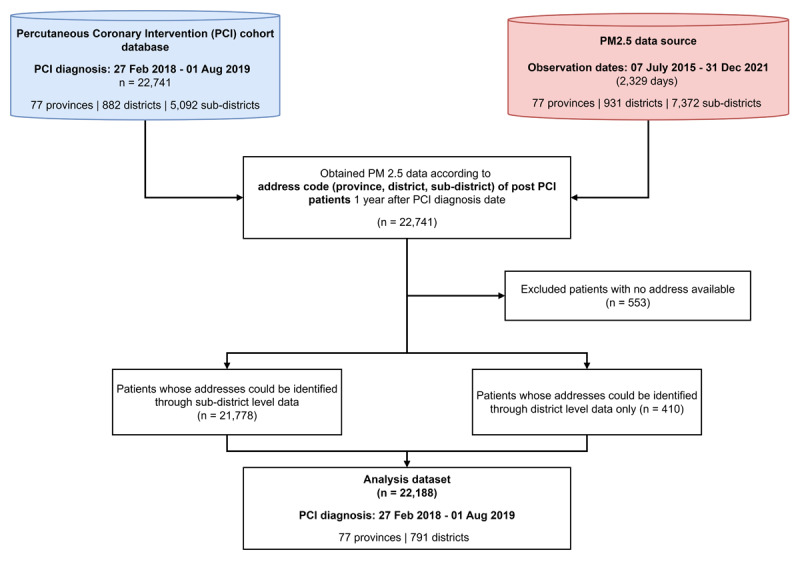
Patient flow chart.

A total of 22,741 participants were enrolled in the Thai PCI registry. For those, their resident areas were successfully linked with PM 2.5 exposure data using strict criteria by sub-district, district, and province. For 963 patients who could not be initially matched at the sub-district level, among these, 410 patients were successfully re-linked by only district and province levels, while the remaining 553 patients (2.43% of the cohort) were excluded from the final analysis. Consequently, 22,188 patients (97.57%) across all 77 provinces in Thailand were included in the study (Figure 1).

The baseline characteristics of the cohort are presented in Table 1. For 22,188 patients, 6,382 patients showed an incidence rate of 2.85 (95% CI: 2.78, 2.92)/100-patients/month. Nearly half of them were elderly (47.6%), with a mean age of 64.2 ± 11.7 years, and the majority were men (68.9%), obese (60.5%), and diagnosed with HT (67.2%), hyperlipidemia (65.1%), CKD stage I/II (67.3%), and ACS (58.1%). Pre-existing cardiovascular conditions were present in a minority of patients, with the most common being previous PCI (29.5%), followed by HF (12.2%), cardiogenic shock (7.9%), stroke (5.7%), and arrhythmia (4.8%). Approximately half of them (45.8%) were followed up during the COVID-19 pandemic period (2020–2022).

**Table 1:** Baseline characteristics of patients stratified by major adverse cardiac and cerebrovascular events (MACCE) (n = 22,188).


VARIABLE	OVERALL MEAN (SD)	NO MACCE (n = 15,806)	MACCE* (n = 6,382)	P-VALUE

PM2.5 (μg/m^3^)				0.555^#^

Mean (SD)	3.28 (9.94)	3.37 (10.45)	3.04 (8.52)	

Median (Q1, Q3)	0.26 (0, 2.29)	0.26 (0, 2.25)	0.28 (0, 2.38)	

Quarters, n (%)				0.623

Q1 (January to March)	5,227 (23.56)	3,761 (71.95)	1,466 (28.05)	

Q2 (April to June)	5,579 (25.14)	3,962 (71.02)	1,617 (28.98)	

Q3 (July to September)	6,367 (28.70)	4,527 (71.10)	1,840 (28.90)	

Q4 (October to December)	5,015 (22.60)	3,556 (70.91)	1,459 (29.09)	

Age group, n (%)				<0.001

< 65	11,620 (52.4)	8,688 (54.9)	2,932 (45.9)	

≥ 65	10,568 (47.6)	7,118 (45.0)	3,450 (54.1)	

Gender, n (%)				<0.001

Female	6,882 (31.0)	4,757 (30.1)	2,125 (33.3)	

Male	15,306 (68.9)	11,049 (69.9)	4,257 (66.7)	

BMI class, n (%)				<0.001

<18.0 Kg/m^2^	1,446 (6.5)	925 (5.9)	521 (8.2)	

18.0–22.9 Kg/m^2^	7,314 (32.9)	5,086 (32.2)	2,228 (34.9)	

23.0–24.9 Kg/m^2^	4,898 (22.1)	3,554 (22.5)	1,344 (21.1)	

25.0–29.9 Kg/m^2^	6,555 (29.5)	4,823 (30.5)	1,732 (27.1)	

≥ 30.0 Kg/m^2^	1,975 (8.9)	1,418 (8.9)	557 (8.7)	

Diabetes mellitus, n (%)				<0.001

Yes	9,814 (44.2)	6,555 (41.5)	3,259 (51.1)	

No	12,374 (55.8)	9,251 (58.5)	3,123 (48.9)	

Hypertension, n (%)				<0.001

Yes	14,900 (67.2)	10,241 (64.8)	4,659 (73.0)	

No	7,288 (32.8)	5,565 (35.2)	1,723 (26.9)	

Dyslipidemia, n (%)				<0.001

Yes	14,450 (65.1)	10,054 (63.6)	4,396 (68.9)	

No	7,738 (34.9)	5,752 (36.4)	1,986 (31.1)	

Chronic kidney disease, n (%)				<0.001

Yes	7,250 (32.7)	4,554 (28.8)	2,696 (42.2)	

No	14,938 (67.3)	11,252 (71.2)	3,686 (57.8)	

Family history of premature CAD, n (%)				0.160

Yes	2,019 (9.1)	1,411 (8.9)	608 (9.5)	

No	20,169 (90.9)	14,395 (91.1)	5,774 (90.5)	

Prior myocardial infarction, n (%)				0.217

Yes	5,214 (23.5)	3,679 (23.3)	1,535 (24.1)	

No	16,974 (76.5)	12,127 (76.7)	4,847 (75.9)	

Cerebrovascular disease, n (%)				<0.001

Yes	1,264 (5.7)	764 (4.8)	500 (7.8)	

No	20,924 (94.3)	15,042 (95.2)	5,882 (92.2)	

Prior PCI, n (%)				<0.001

Yes	6,538 (29.5)	4,867 (30.8)	1,671 (26.2)	

No	15,650 (70.5)	10,939 (69.2)	4,711 (73.8)	

Prior CABG, %				0.164

Yes	362 (1.6)	246 (1.6)	116 (1.8)	

No	21,826 (98.4)	15,560 (98.4)	6,266 (98.2)	

Smoking, n (%)				<0.001

Never	9,963 (44.9)	6,989 (44.2)	2,974 (46.6)	

Former	7,051 (31.8)	5,012 (31.7)	2,039 (31.9)	

Current	5,174 (23.3)	3,805 (24.1)	1,369 (21.5)	

Heart Failure, n (%)				<0.001

Yes	2,714 (12.2)	1,655 (10.5)	1,059 (16.6)	

No	19,474 (87.8)	14,151 (89.5)	5,323 (83.4)	

Cardiogenic Shock, n (%)				<0.001

Yes	1,763 (7.9)	1,133 (7.2)	630 (9.9)	

No	20,425 (92.1)	14,673 (92.8)	5,752 (90.1)	

Arrhythmia, n (%)				<0.001

Yes	1,056 (4.8)	647 (4.1)	409 (6.4)	

No	21,132 (95.2)	15,159 (95.9)	5,973 (93.6)	

Types of CAD, n (%)				<0.001

Stable	9,298 (41.9)	6,812 (43.1)	2,486 (38.9)	

NSTEMI/Unstable Angina	6,630 (29.9)	4,567 (28.9)	2,063 (32.3)	

STEMI	6,260 (28.2)	4,427 (28.0)	1,833 (28.7)	

Lesion location, n (%)				<0.001

Left Main	2,627 (11.8)	1,711 (10.8)	916 (14.4)	

Others vessel disease	19,561 (88.2)	14,095 (89.2)	5,466 (85.6)	

Number of stent(s) used, n (%)				<0.001

0 stent	1,728 (7.8)	1,017 (6.4)	711 (11.1)	

1 stent	11,388 (51.3)	8,613 (54.5)	2,775 (43.5)	

2 stents	6,264 (28.2)	4,297 (27.2)	1,967 (30.8)	

≥ 3 stents	2,808 (12.7)	1,879 (11.9)	929 (14.6)	

Number of lesion(s), n (%)				0.162

1 lesion	17,626 (79.4)	12,597 (79.7)	5,029 (78.8)	

2 lesions	3,826 (17.2)	2,678 (16.9)	1,148 (17.9)	

≥3 lesions	736 (3.3)	531 (3.4)	205 (3.2)	

Follow up time during COVID-19 period				<0.001

Yes^+^	10,156 (45.8)	7,830 (49.5)	2,326 (36.4)	

No^++^	12,032 (54.2)	7,976 (50.5)	4,056 (63.6)	


Abbreviations: BMI: body mass index; CAD: coronary artery disease; CVA: cerebrovascular accident; PCI: percutaneous coronary intervention; CABG: coronary artery bypass graft surgery; NSTEMI: non-ST-segment elevation myocardial infarction; UA: unstable angina; ST-segment elevation myocardial infarction; LM: left main; MACCE: major adverse cardiac and cerebrovascular events. *MACCE occurrence at least one time during one-year follow-up; # Wilcoxon’s rank-sum test; + During years 2020–2022; ++ During years 2018–2020.

**Figure 2:**
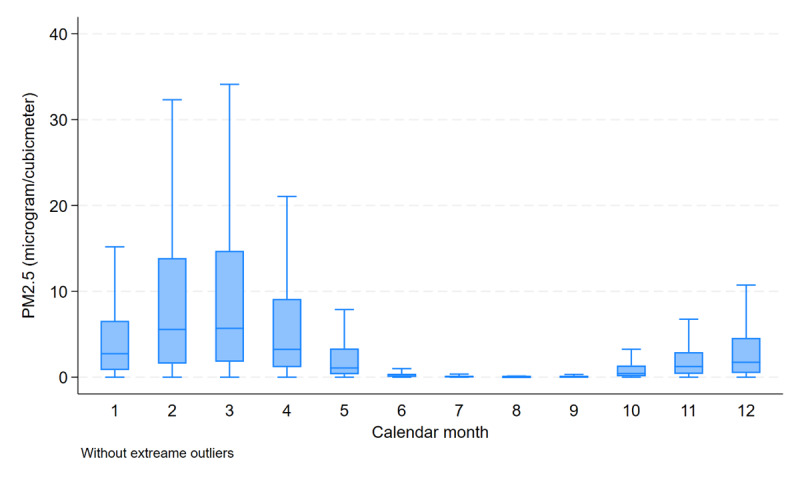
Monthly average concentration of PM2.5 exposure of Thai adults who underwent percutaneous coronary intervention during 2018–2019. Abbreviations: PM2.5: particulate matter with diameter of less than 2.5 micron.

The distribution and seasonal variation of PM2.5 concentrations are presented in Figure 2 and Supplementary Table 1. The lowest PM2.5 levels were observed during the rainy season (July to September), with a median (IQR) concentration of 0.016 (0.000–0.107) µg/m^3^. This reduction is likely attributable to the combined effects of heavy rainfall and increased wind activity, which facilitate the dispersion and wet scavenging of aerosol particles from the atmosphere. In contrast, the highest PM2.5 concentrations occurred between January and March, with a median (IQR) of 4.247 (1.326, 11.610), corresponding to Thailand’s dry season and peak period of agricultural burning. This seasonal peak was particularly prominent in the northern and northeastern regions, as illustrated by the spatial distribution of annual average PM2.5 levels by province in Supplementary Figure 1.

During 252,110.8 person-months of follow-up, a total of 7,163 MACCE events were observed, corresponding to an incidence rate of 0.028 per person-month (95% CI: 0.027, 0.029). Univariable Weibull survival regression indicated that an increase in PM2.5 by 1 μg/m^3^ was associated with an HR of 0.996 (95% CI: 0.994, 0.999, p = 0.012), suggesting a paradoxical protective effect. Additionally, seasonal variations in PM2.5 levels, represented by calendar quarters, tended to show a protective trend compared with the lowest PM2.5 quarter 3 (Q3), although this was not statistically significant. Other factors significantly associated with MACCE, as detailed in Table 2, included advanced age (≥ 65 years), female sex, underweight status, DM, HT, DLP, CKD, CVA, prior CABG, HF, cardiogenic shock, arrhythmia, ACS, and LM coronary artery disease.

**Table 2:** Univariable and multivariable multi-level Cox PH models of risk factors for major adverse cardiac and cerebrovascular events (MACCE), stratified by PM2.5 exposure.


VARIABLE	PERSON-MONTH	INCIDENCE RATE	HR (95%CI)	P-VALUE	ADJUSTED HR (95%CI)	P-VALUE

PM2.5 (μg/m^3^)^#^	–	–	1.00 (0.99, 1.00)	0.012	1.45 (1.37, 1.54)	<0.001

Quarters						

Q1	63,000.0	0.028 (0.026, 0.029)	0.95 (0.89, 1.01)	0.108	1.18 (1.09, 1.27)	<0.001

Q2	60,966.9	0.028 (0.026, 0.029)	0.94 (0.88, 1.01)	0.078	1.17 (1.09, 1.28)	<0.001

Q3	63,423.5	0.031 (0.029, 0.032)	1	–	1	–

Q4	64,046.2	0.028 (0.027, 0.029)	0.96 (0.90, 1.03)	0.252	0.97 (0.90, 1.03)	0.322

Age (Y)						

< 65	134,696.5	0.024 (0.023, 0.025)	1	<0.001	1	<0.001
	
≥ 65	116,740.1	0.033 (0.032, 0.034)	1.36 (1.30, 1.43)		1.13 (1.07, 1.19)	

Gender						

Female	76,705.9	0.032 (0.030, 0.033)	1.16 (1.11, 1.22)	<0.001	–	–

Male	174,730.7	0.027 (0.026, 0.028)	1			

BMI (Kg/m^2^)						

<18.0	15,191.9	0.040 (0.037, 0.043)	1.29 (1.18, 1.42)	<0.001	1.27 (1.16, 1.39)	<0.001

18.0–22.9	81,714.8	0.031 (0.030, 0.032)	1	1	1	–

23.0–24.9	55,967.6	0.027 (0.026, 0.028)	0.87 (0.82, 0.93)	<0.001	0.90 (0.84, 0.96)	0.001

25.0–29.9	75,772.0	0.025 (0.024, 0.027)	0.83 (0.78, 0.88)	<0.001	0.85 (0.80, 0.90)	<0.001

≥ 30.0	22,790.3	0.027 (0.025, 0.029)	0.87 (0.80, 0.95)	0.003	0.87 (0.79, 0.95)	0.003

Diabetes mellitus, n (%)						

Yes	109,785.6	0.034 (0.033, 0.035)	1.38 (1.31, 1.44)	<0.001	1.19 (1.13, 1.25)	<0.001
	
No	141,651.0	0.024 (0.024, 0.025)	1		1	

Hypertension, n (%)						

Yes	168,150.1	0.031 (0.030, 0.032)	1.35 (1.28, 1.43)	<0.001	1.18 (1.11, 1.26)	<0.001
	
No	83,286.5	0.023 (0.022, 0.024)	1		1	

Dyslipidemia, n (%)						

Yes	164,958.8	0.030 (0.029, 0.031)	1.16 (1.11, 1.23)	<0.001	1.13 (1.07, 1.20)	<0.001
	
No	86,477.8	0.025 (0.024, 0.026)	1		1	

CKD, n (%)						

Yes	78,128.0	0.040 (0.038, 0.041)	1.67 (1.59, 1.75)	<0.001	1.39 (1.32, 1.47)	<0.001
	
No	173,308.6	0.023 (0.023, 0.024)	1		1	

CVA, n (%)						

Yes	13,681.0	0.043 (0.040, 0.047)	1.52 (1.40, 1.66)	<0.001	1.29 (1.18, 1.41)	<0.001
	
No	237,755.6	0.028 (0.027, 0.028)	1		1	

Prior PCI, n (%)						

Yes	75,238.6	0.025 (0.024, 0.026)	0.80 (0.76, 0.85)	<0.001	0.79 (0.74, 0.84)	<0.001
	
No	176,198.0	0.030 (0.029, 0.031)	1		1	

Prior CABG, n (%)						

Yes	4,045.0	0.035 (0.029, 0.041)	1.19 (1.00, 1.41)	0.045	–	–

No	247,391.6	0.028 (0.028, 0.029)	1			

Smoking, n (%)						

Never	112,087.2	0.030 (0.029, 0.031)	1	–	–	–

Former	80,709.3	0.028 (0.027, 0.029)	0.94 (0.89, 0.99)	0.018		

Current	58,640.1	0.026 (0.025, 0.027)	0.88 (0.82, 0.93)	<0.001		

Heart Failure, n (%)						

Yes	27,106.2	0.044 (0.042, 0.047)	1.70 (1.60, 1.81)	<0.001	1.51 (1.38, 1.65)	<0.001
	
No	224,330.4	0.027 (0.026, 0.027)	1		1	

Cardiogenic Shock, n (%)						

Yes	17,332.7	0.040 (0.037, 0.043)	1.49 (1.37, 1.61)	<0.001	0.81 (0.72, 0.91)	<0.001
	
No	234,103.9	0.028 (0.027, 0.028)	1		1	

Arrhythmia, n (%)						

Yes	10,743.4	0.042 (0.039, 0.046)	1.51 (1.37, 1.67)	<0.001	1.19 (1.07, 1.32)	0.002
	
No	240,693.2	0.028 (0.027, 0.029)	1		1	

Types of CAD, n (%)						

Stable	107,637.5	0.026 (0.025, 0.027)	1	–	1	–

NSTEMI/UA	74,898.2	0.031 (0.029, 0.032)	1.23 (1.17, 1.31)	<0.001	1.08 (1.02, 1.15)	0.014

STEMI	68,900.9	0.030 (0.029, 0.031)	1.21 (1.14, 1.28)	<0.001	1.15 (1.07, 1.23)	<0.001

Lesion location, n (%)						

LM	28,876.7	0.036 (0.034, 0.038)	1.29 (1.21, 1.38)	<0.001	1.12 (1.05, 1.20)	0.001
	
Others	222,559.9	0.028 (0.027, 0.028)	1		1	

No. of stent(s) used, n (%)						

0 stent	18,666.7	0.044 (0.041, 0.047)	1	–	1	–

1 stent	130,115.8	0.024 (0.023, 0.024)	0.54 (0.50, 0.59)	<0.001	0.58 (0.54, 0.63)	<0.001

2 stents	71,081.4	0.031 (0.030, 0.033)	0.72 (0.66, 0.78)	<0.001	0.74 (0.68, 0.80)	<0.001

≥ 3 stents	31,572.8	0.033 (0.031, 0.035)	0.75 (0.68, 0.82)	<0.001	0.76 (0.69, 0.84)	<0.001

Follow up time during COVID-19 period						

Yes*	37,794.1	0.009 (0.008, 0.010)	0.42 (0.38, 0.47)	<0.001	0.39 (0.35, 0.44)	<0.001

No**	213,642.5	0.032 (0.031, 0.033)	1	–	1	


Abbreviations: MACCE: major adverse cardiac and cerebrovascular events; BMI: body mass index; CKD: chronic kidney disease; CAD: coronary artery disease; MI: myocardial infarction; CVA: cerebrovascular accident; PCI: percutaneous coronary intervention; CABG: coronary artery bypass graft surgery; STEMI: ST-segment elevation myocardial infarction; NSTEMI: non-ST-segment elevation myocardial infarction; UA: unstable angina; LM: left main. Q1 (January to March), Q2 (April to June), Q3 (July to September), and Q4 (October to December), which are not analyzed with interaction effect. ^#^Continuous variable, *During years 2020–2022, **During years 2018–2019.

The final multivariable model showed that incorporating the interaction effect between PM2.5 and the quarter of the year improved the overall model fit (LR Chi-squared = 165.19, p < 0.001), so this study also analyzed the adjusted HR of PM2.5 exposure (per 1 µg/m^3^ increase) and the quarter of the year for a one-year MACCE outcome. Their adjusted HRs are presented in Table 2.

**Figure 3:**
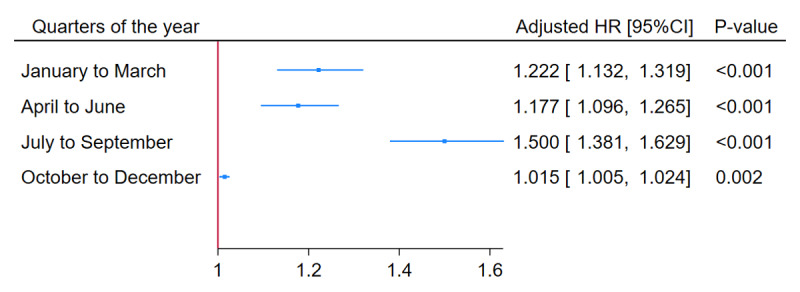
Adjusted hazard ratio of PM2.5 exposure (per 1 µg/m^3^ increase) and quarters of the year for one-year major adverse cardiac and cerebrovascular (MACCE) outcome in Thai adults underwent PCI during 2018–2019.

The impact of PM2.5 (per 1 µg/m^3^ increase) in each quarter, along with their interaction effects, was as follows: 1.014 (95% CI: 1.005, 1.024) in Q4, rising to 1.223 (95% CI: 1.133, 1.320) in Q1, slightly decreasing to 1.178 (95% CI: 1.096, 1.266) in Q2, and reaching 1.500 (95% CI: 1.380, 1.628) in Q3, as illustrated in the forest plot in Figure 3.

In subgroup analyses stratified by CAD subtype and regional wildfire activity (Supplementary Tables 2 and 3), PM2.5 exposure was consistently associated with increased risk of MACCE, in line with the main analysis. Across CAD subtypes, the adjusted HRs per μg/m^3^ increase in PM2.5 were 1.62 (95% CI: 1.46, 1.80) for stable CAD, 1.38 (95% CI: 1.26, 1.51) for NSTEMI/unstable angina, and 1.40 (95% CI: 1.25, 1.58) for STEMI (all p < 0.001). Quarter-specific were observed in stable CAD and NSTEMI/unstable angina, indicating seasonal modulation, whereas no significant quarter effects were detected in the STEMI subgroup, despite a significant overall association with PM2.5. When stratified by wildfire region, the magnitude and temporal pattern of PM2.5-associated risk were similar in high- and low-wildfire areas, with significant associations observed.

## Discussion

This study evaluated the prognostic effect of the PM2.5 exposure of each season on the composite MACCE outcome of patients who received PCI. During the one-year follow-up, a higher level of PM2.5 exposure was associated with MACCE outcomes after discharge, particularly for patients presenting with ACS. In univariable analyses, the association between PM2.5 exposure and MACCE appeared modest, reflecting the influence of seasonal and patient-level factors correlated with both exposure and outcome. However, after comprehensive multivariable adjustment, higher PM2.5 exposure was associated with an increased risk of MACCE after discharge. Notably, this association demonstrated seasonal heterogeneity and confounding effects. The overall pattern of association remained robust across subgroup analyses. It should be noted that dose–response analyses suggested a generally monotonic increase in MACCE risk with higher PM2.5 exposure, without evidence of a clear threshold effect. This pattern may reflect limited exposure contrast and the unavoidable measurement error inherent in ambient air pollution assessment.

The findings of this study correspond with the results from previous studies. In a retrospective cohort study of patients with a history of acute MI or stroke, the mean annual PM2.5 exposure was associated with an increased CV mortality (26). Furthermore, a prior meta-analysis conducted by Stacey E. et al. (14) demonstrated that a 10-µg/m^3^ increase in long-term PM2.5 exposure was significantly associated with an increased risk of CV death and incidence of MI and stroke. In a recent study (27) of U.S. veterans who received elective PCI, long-term exposure to PM2.5 was associated with risk of MACE. However, no study has evaluated the effect of PM2.5 exposure to CV events in patients with ACS who underwent emergency or urgent PCI during the COVID-19 pandemic. PM2.5 exposure may be associated with the increased lethality of COVID-19 infection through its effect on CVD (32). In addition, a statistically significant association was found between daily COVID-19 confirmed cases and rainfall in Bangkok (33), and the summer and monsoon seasons showed a statistically significant positive association with the trend in the number of COVID-19 cases. In contrast, this study demonstrated a protective association during the COVID-19 pandemic period (adjusted HR 0.39; 95% CI 0.35–0.44). This finding should not be interpreted as a direct protective effect of COVID-19 itself. Rather, the pandemic period variable was included to account for healthcare system disruptions during this time—such as constrained hospital capacity, altered care pathways, and workforce reallocation—rather than the biological effects of SARS-CoV-2 infection. Accordingly, the pandemic indicator was treated as an adjustment covariate in all multivariable models to control for its potential confounding influence on MACCE risk.

The association between PM2.5 exposure and worse CV outcomes in patients with ACS could be explained by several biological mechanisms (21), including inflammation, oxidative stress, autonomic imbalance of sympathetic tone, endothelial barrier disruption and/or dysfunction, systemic vascular dysfunction, central nervous system effects on metabolism, and epigenomic changes. The air pollution-driven increase in the membrane receptor angiotensin 2-converting enzyme (ACE2) represents an important determinant of the susceptibility of human alveolar epithelial cells to SARS-CoV-2 infection and stands at the base of a synergistic mechanism involving PM and viral particles to promote the pro-inflammatory response (34).

Quarter-specific analyses revealed temporal heterogeneity in the PM2.5–MACCE association, with the strongest effect observed in the third quarter (July–September), corresponding to Thailand’s rainy season. Although rainfall generally reduces ambient PM2.5 concentrations, our findings suggest that cardiovascular risk is not determined solely by exposure mass. Seasonal differences in PM2.5 composition may also contribute; PM2.5 in the first and second quarters is largely driven by biomass and agricultural burning, whereas rainy-season exposures are lower in concentration but may differ in chemical and physical characteristics. However, comparable PM2.5–MACCE associations across high- and low-wildfire regions indicate that seasonal heterogeneity is unlikely to be explained solely by geographic variation in burning-related exposure.

Instead, seasonal modifiers such as lower temperatures, higher humidity, and increased meteorological variability during the rainy season may enhance population susceptibility and modify PM2.5 toxicity (35). Previous studies (36,37) have demonstrated that fluctuations in temperature and humidity are significantly associated with the occurrence of myocardial infarction, sudden cardiac death, and overall cardiovascular mortality. The persistence of a strong association in Q3 despite lower PM2.5 levels supports the notion that periods of reduced ambient concentrations may still confer substantial cardiovascular risk due to heightened physiological vulnerability or changes in particle toxicity. These findings highlight the importance of incorporating seasonal context when evaluating air pollution–related cardiovascular effects. Moreover, potential contributing factors not explored in this study may include the presence of microplastics and nanoplastics, which are increasingly detected in PM2.5 (38), particularly during the rainy season (39). Emerging evidence suggests that microplastics in the circulatory system may promote thrombogenesis through size-dependent biological pathways, platelet aggregation, and hemolytic activity (40).

In subgroup analyses, the association between PM2.5 exposure and MACCE remained robust across CAD phenotypes and regional wildfire activity, reinforcing the internal consistency of the main findings. Notably, the strongest association was observed among patients with stable CAD, suggesting that chronic atherosclerotic disease may be particularly susceptible to the cumulative vascular and inflammatory effects of sustained ambient particulate exposure. The presence of quarter-specific effects in stable CAD and NSTEMI/unstable angina—but not in STEMI—indicates potential seasonal modulation of PM2.5-related risk in non–ST-elevation presentations, possibly reflecting interactions between temporal variations in pollution sources, systemic inflammatory responses, and patterns of healthcare utilization. In contrast, the absence of detectable seasonal effects in STEMI, despite a significant overall association, aligns with prior evidence that ST-elevation events are more influenced by acute exposure peaks rather than longer-term seasonal variation in PM2.5 levels.

This study had some limitations. This study was a retrospective cohort analysis that could not collect and evaluate the variation of some covariates on clinical outcomes over the follow-up period, e.g., blood sugar, HbA1C, blood pressure level, troponin, medication, medical adherence, concomitant inflammation, malignancy, and genetic factors, because of our limited data. Other confounders such as particle toxicity, climatic factors, or population susceptibility might also play an important role in studying PM2.5 and environmental factors. Second, the PCI registry did not distinguish between NSTEMI and unstable angina, which differ in long-term prognosis; therefore, we were unable to evaluate potential differences in PM2.5-associated risk between these two ACS subtypes. Additionally, PM2.5 exposure was estimated using monthly averages at the subdistrict level, which may not accurately reflect individual-level exposure due to variability in personal behaviors, use of protective measures, and potential migration across residential areas. Furthermore, 963 patients (4.23%) could not be linked to sub-district–level exposure data; however, 410 of these patients were successfully re-linked at the district level. Consequently, 553 patients (2.43%) were excluded from the final analysis. Although this exclusion may introduce some selection bias, the relatively small proportion of missing data supports the appropriateness of a complete-case analysis.

This study had several strengths. This was the first multicenter study to analyze the prognostic effect of PM2.5 exposure for MACCE outcomes in a large population who underwent PCI. Multiple risk factors for coronary artery disease have been established, including age, gender, a family history of heart disease, HT, DM, hyperlipidemia, physical inactivity, unhealthy eating, and smoking tobacco. Air pollution, genes, immunity, metabolic, and lipid-related actors have emerged as risk factors for CVD (21,41). As such, this study included both the identified risk factors and PM2.5 exposure as confounding factors in the multivariable analysis, and it still found a significant correlation between PM2.5 exposure and the composite MACCE outcome. Likewise, this study also analyzed a subset of the ACS population and found a similar effect of PM2.5 exposure. Further prospective studies focusing on other air pollutants such as ozone, carbon monoxide, nitrogen oxides, and sulfur dioxides should be performed in patients with and without CVD. In addition, further studies should investigate the combined effects of PM2.5, microplastics, and nanoplastics on human health, particularly on the cardiovascular system as well as other related physiological systems. Such investigations may require more complex analyses, especially regarding interactions with regional variations in temperature and humidity, which can differ significantly across the globe. Additionally, governments should implement comprehensive strategies to address climate variability, taking into account the distinct characteristics of each season. For instance, during the rainy season, enhanced monitoring of additional environmental pollutants may be necessary even when PM levels are relatively low in order to provide early warnings and enable the public to take appropriate protective measures.

## Conclusion

This study provides the first multicenter evidence regarding the prognostic effect of PM2.5 exposure on MACCE in a broad population of PCI-treated patients. The independent association between increased PM2.5 levels and adverse cardiovascular outcomes after discharge was robust to subgroup stratification and persistent despite seasonal heterogeneity. These results underscore the importance of accounting for environmental factors in post-PCI prognosis and the urgent need for public health policies that focus on reducing air pollution to improve health outcomes and reduce the burden of cardiovascular disease.

## Data Accessibility Statement

The data that support the findings of this study are available from the co-author, [N.S.], upon reasonable request.

## Additional File

The additional file for this article can be found as follows:

10.5334/gh.1539.s1 Supplementary Files 1. Supplementary Tables 1 to 3 and Figure 1.
