# Supplementary material for: Fine Particulate Matter Exposure and Risk of Major Adverse Cardiac and Cerebrovascular Events (MACCE) in Post-Percutaneous Coronary Intervention (PCI) Patients: A Thai PCI Registry-Based Cohort Study
Source: Glob Heart. 2026 Mar 17;21(1):23. doi: 10.5334/gh.1539 (PMC13004060; doi:10.5334/gh.1539)

Supplemental Legend

Supplementary Table 1 Monthly, Quarterly and Yearly averages of PM2.5 and MACCE outcome of patients who underwent percutaneous coronary intervention

Supplementary Table 2 Subgroup analyses of the association between PM2.5 exposure and MACCE stratified by coronary artery disease subtype (stable CAD, NSTEMI/unstable angina, and STEMI).

Supplementary Table 3 Subgroup analyses of the association between PM2.5 exposure and MACCE stratified by regional wildfire activity (high vs low wildfire regions).

Supplementary Figure 1 Distribution of annual average of PM2.5 levels across Thailand by provinces by applying an upper limit of 30 μg/m³

Supplementary Table 1 Monthly, Quarterly and Yearly averages of PM2.5 and MACCE outcome of patients who underwent percutaneous coronary intervention

Time	Overall Median (IQR)	No MACCE Median (IQR)	MACCE at least once Median (IQR)	P-value*
Monthly				
January	2.731 (0.821, 6.568)	2.736 (0.785, 6.612)	2.717 (0.889, 6.405)	0.780
February	5.553 (1.575, 13.879)	5.586 (1.549, 13.996)	5.424 (1.684, 13.566)	0.676
March	5.687 (1.795, 14.722)	5.757 (1.800, 14.992)	5.505 (1.778, 13.815)	0.029
April	3.235 (1.173, 9.125)	3.338 (1.187, 9.621)	3.000 (1.130, 7.941)	<0.001
May	1.079 (0.327, 3.347)	1.097 (0.328, 3.445)	1.026 (0.324, 3.062)	0.004
June	0.123 (0.020, 0.411)	0.127 (0.020, 0.426)	0.116 (0.019, 0.381)	0.019
July	0.043 (0.001, 0.146)	0.044 (0.001, 0.151)	0.041 (0.002, 0.131)	0.047
August	0.000 (0.000, 0.054)	0.000 (0.000, 0.056)	0.000 (0.000, 0.050)	0.371
September	0.005 (0.000, 0.133)	0.005 (0.000, 0.132)	0.007 (0.000, 0.137)	0.100
October	0.414 (0.104, 1.364)	0.404 (0.101, 1.338)	0.449 (0.110, 1.428)	0.007
November	1.252 (0.376, 2.929)	1.226 (0.356, 2.894)	1.345 (0.440, 3.008)	<0.001
December	1.742 (0.482, 4.583)	1.767 (0.466, 4.680)	1.683 (0.538, 4.312)	0.302
Quarterly				
1	4.247 (1.326, 11.61)	4.290 (1.313, 11.804)	4.136 (1.367, 11.111)	0.124
2	0.842 (0.169, 3.39)	0.858 (0.171, 3.485)	0.802 (0.162, 3.130)	<0.001
3	0.016 (0.000, 0.107)	0.016 (0.000, 0.109)	0.016 (0.000, 0.101)	0.693
4	0.993 (0.238, 2.764)	0.968 (0.228, 2.773)	1.066 (0.269, 2.728)	<0.001
Yearly	0.665 (0.069, 3.379)	0.659 (0.069, 3.430)	0.682 (0.067, 3.243)	0.0878

* Wilcoxson's rank-sum test

MACCE: major adverse cardiac and cerebrovascular events

Supplementary Table 2 Subgroup analyses of the association between PM2.5 exposure and major adverse cardiac and cerebrovascular events stratified by coronary artery disease subtype (stable CAD, NSTEMI/unstable angina, and STEMI)

Variables	Stable n = 9,298 (41.91%)		NSTEMI/Unstable angina n = 6,630 (29.88%)		STEMI n = 6,260 (28.21%)	
	**Adjusted HR**	**p-value**	**Adjusted HR**	**p-value**	**Adjusted HR**	**p-value**
PM2.5 (μg/m^3)	1.62 (1.46, 1.80)	<0.001	1.38 (1.26, 1.51)	<0.001	1.40 (1.25, 1.58)	<0.001
Quarters						
Q1	1.32 (1.17, 1.49)	<0.001	1.16 (1.02, 1.31)	0.024	1.06 (0.93, 1.22)	0.387
Q2	1.20 (1.08, 1.35)	0.001	1.24 (1.09, 1.40)	0.001	1.09 (0.95, 1.24)	0.204
Q3	1	-	1	-	1	-
Q4	1.03 (0.92, 1.15)	0.607	0.90 (0.79, 1.02)	0.101	0.96 (0.84, 1.09)	0.496
PM2.5 and Q effects						
Q1	1.32 (1.17, 1.48)	<0.001	1.16 (1.03, 1.32)	0.018	1.07 (0.93, 1.22)	0.343
Q2	1.18 (1.06, 1.31)	0.003	1.19 (1.05, 1.33)	0.004	1.06 (0.93, 1.20)	0.387
Q3	1.62 (1.46, 1.80)	<0.001	1.38 (1.26, 1.51)	<0.001	1.40 (1.25, 1.58)	<0.001
Q4	1.05 (0.94, 1.17)	0.365	0.91 (0.80, 1.02)	0.109	0.97 (0.85, 1.09)	0.581

Supplementary Table 3. Subgroup analyses of the association between PM2.5 exposure and major adverse cardiac and cerebrovascular events stratified by regional wildfire activity (high vs low wildfire regions).

Variables	High wildfire n = 13,176 (59.38%)		Low wildfire n = 9,012 (40.62%)	
	**Adjusted HR**	**p-value**	**Adjusted HR**	**p-value**
PM2.5 (μg/m^3)	1.43 (1.34, 1.52)	<0.001	1.44 (1.20, 1.73)	<0.001
Quarters				
Q1	1.20 (1.09, 1.31)	<0.001	1.17 (1.04, 1.33)	0.009
Q2	1.25 (1.14, 1.36)	<0.001	1.09 (0.97, 1.23)	0.167
Q3	1	-	1	-
Q4	0.95 (0.87, 1.03)	0.225	1.01 (0.90, 1.13)	0.914
PM2.5 and Q effects				
Q1	1.20 (1.09, 1.31)	<0.001	1.18 (1.05, 1.34)	0.005
Q2	1.20 (1.11, 1.31)	<0.001	1.06 (0.94, 1.19)	0.351
Q3	1.43 (1.34, 1.52)	<0.001	1.44 (1.20, 1.73)	<0.001
Q4	0.96 (0.88, 1.04)	0.290	1.03 (0.92, 1.15)	0.598

Supplementary Figure 1 Distribution of annual average of $PM_{2.5}$ levels across Thailand by provinces by applying an upper limit of 30 µg/m³

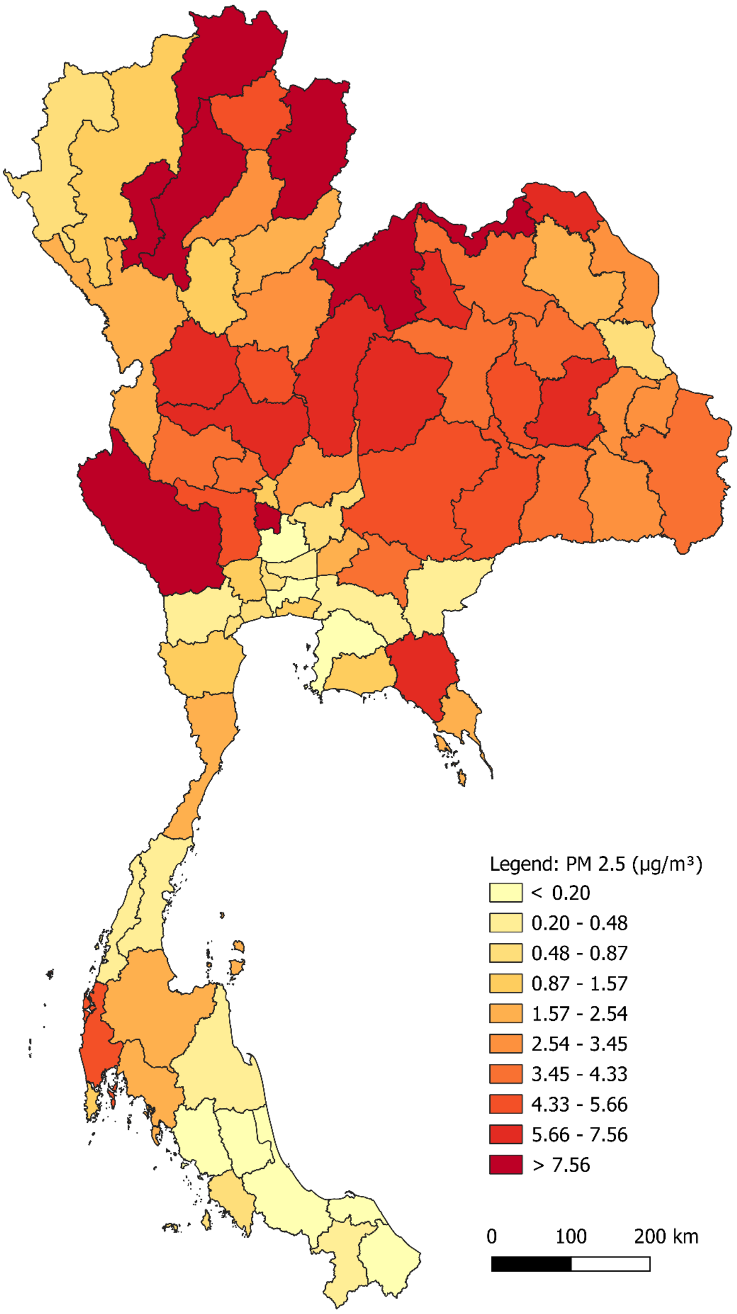

Supplement: Supplementary Files 1. — Supplementary Tables 1 to 3 and Figure 1. [file gh-21-1-1539-s1.pdf]
